# A Systematic Review and Meta-Analysis to Assess Patient-Reported Outcomes after Lung Cancer Surgery

**DOI:** 10.1155/2013/789625

**Published:** 2013-11-03

**Authors:** Sayf Gazala, Jean-Sébastien Pelletier, Dale Storie, Jeffrey A. Johnson, Demetrios J. Kutsogiannis, Eric L. R. Bédard

**Affiliations:** ^1^Depratment of Surgery, University of Alberta, Edmonton, AB, Canada T6G 2B7; ^2^416 Community Services Center, Royal Alexandra Hospital, 10240 Kingsway Avenue, Edmonton, AB, Canada T6G 2B7; ^3^John W. Scott Health Sciences Library, University of Alberta, Edmonton, AB, Canada T6G 2R7; ^4^School of Public Health, University of Alberta, Edmonton, AB, Canada T6G 1C9; ^5^Division of Critical Care Medicine, Faculty of Medicine and Dentistry, University of Alberta, Edmonton, AB, Canada T6G 2B7

## Abstract

The main objective of this review was to systematically review, assess, and report on the studies that have assessed health related quality of life (HRQOL) after VATS and thoracotomy for resection of lung cancer. We performed a systematic review of six databases. The Downs and Black tool was used to assess the risk of bias. Five studies were included. In general, patients undergoing VATS have a better HRQOL when compared to thoracotomy; however, there was a high risk of bias in the included studies. The consistent use of a lung cancer specific questionnaire for measuring HRQOL after surgery is encouraged.

## 1. Introduction

Lung cancer accounts for about 15% of all cancers and for about 28% of all cancer deaths. More than 400,000 people alive today in the United States have been diagnosed with lung cancer [1]. Surgical removal, via thoracotomy or VATS, in the form of anatomical resection depending on the location of the tumor is the preferred treatment approach for patients with early stage NSCLC (i.e., stages I and II) [2, 3]. The VATS approach involves anatomical hilar dissection, with individual ligation of lobar vessels and bronchus as well as hilar lymph node dissection or sampling, without rib spreading [4].

Policymakers and health care professionals have recognized the importance of measuring health related quality of life (HRQOL). This is occurring for a variety of reasons, which include but is not limited to improved clinical care and economics [5]. Over the last few decades, several studies have been conducted to assess the HRQOL of patients with lung cancer after surgery using different measures, comparing different criteria, and reaching various conclusions. No systematic review has been conducted to compare the results of the studies that have assessed HRQOL after lung cancer surgery.

Two systematic reviews comparing VATS to thoracotomy for the treatment of early stage lung cancer have been conducted looking at the following outcomes: chest tube duration, length of hospital stay, survival, perioperative morbidity, and biological and oncological outcomes. These reviews demonstrated equivalent oncologic outcomes between the two approaches, but favored the VATS approach in all the other outcomes [6, 7].

## 2. Objectives

The main objective of this review was to systematically locate, review, assess, and report on the studies that have assessed HRQOL after VATS and thoracotomy for resection of nonsmall cell lung cancer. Secondary objectives include the assessment of HRQOL in the early versus the late post-operative period between the VATS and the thoracotomy approaches and the assessment of HRQOL based on the post-operative surgical complications. We used three months after surgery as a cutoff for defining the early post-operative period.

## 3. Materials and Methods

A protocol was developed a priori by the reviewers and was registered at PROSPERO database, identifier: CRD42012002159.

### 3.1. Inclusion Criteria

- Study designs: clinical trials and observational studies.
- Participants: patients with surgical resection for early stage NSCLC.
- Intervention: VATS
- Comparison: thoracotomy
- Outcome: HRQOL.

### 3.2. Exclusion Criteria

- Study design: reviews and meta-analysis.
- Population: studies that have included patients with benign diseases, and patients with other types of cancers.
- Intervention/comparison: studies that have pooled data for the VATS and thoracotomy together and studies that have compared surgical resection to other modalities of treatment.
- Outcome: studies that have not included HRQOL as an outcome.

### 3.3. Search Strategy for Identification of Studies

A research librarian (DS) in collaboration with the first author (SG) developed and implemented a search strategy designed to identify evidence relevant to this review (the appendix). Search filters were used to find pertinent English language studies from date of inception to May, 15 2012, inclusively. We systematically searched the following electronic databases: Medline, EMBASE, Scopus, Cochrane Central Register of Controlled Trials, Cochrane Database of Systematic Reviews, Google Scholar, and Health Technology Assessment Database. We also searched the reference lists of included studies and previous reviews in this field. A gray literature search was carried out to identify further studies by examining conference proceedings for the previous five years of the Society of Thoracic Surgeons, the Western Thoracic Surgical Association, and the European Association for Cardio-Thoracic Surgery. Only full manuscripts were included; abstracts were searched to identify other studies.

Two reviewers (Sayf Gazala and Jean-Sébastien Pelletier) carried out the first step of the screening process independently which involved reading the titles and the abstracts using broad criteria. Each study was classified as include, exclude, or unclear. Articles that were classified as “include” or “unclear” by either reviewers were included for full text review.

In the second step of the screening process, two authors (Sayf Gazala and Jean-Sébastien Pelletier) independently assessed each study using a standard form that outlined the predetermined inclusion criteria. All disagreements were resolved via third party adjudication performed by the final author.

The Downs and Black assessment tool was used to assess the methodological quality of the included studies by two reviewers (Sayf Gazala and Jean-Sébastien Pelletier) at the study level [8]. Once again, all disagreements were resolved via third party adjudication performed by a third author (JJ).

### 3.4. Data Extraction, Analysis, and Synthesis

Data extraction was carried out by two reviewers (Sayf Gazala and Jean-Sébastien Pelletier) and independently adjudicated by two other reviewers (Eric L.R. Bédard and Demetrios J. Kutsogiannis) using a pilot data extraction form developed to address the research question. Disagreements were resolved by discussion among the reviewers. 

The data that were extracted included study design, year and country, number of participants, intervention used, surgical procedure performed, outcome(s), study funding, and HRQOL data.

Results of the included studies were grouped based on the different study designs. Then, based on the surgical approach (VATS versus thoracotomy), the data were aggregated and analyzed in the different study designs. Qualitative analysis was carried out to present what have been done in the field and identify gaps in the literature for future research plans.

The mean difference (MD) was used for continuous data with 95% confidence intervals (CI). Where feasible, a meta-analysis was conducted to estimate a pooled weighted mean difference in subscales of commonly applied HRQOL instruments. Pooling was planned a priori when statistical heterogeneity, assessed by the *I* ^2^ statistics, was <50% [9].

We planned two subgroup analyses; the first between early versus late assessment of HRQOL between the VATS and the thoracotomy approaches using less than or equal to three months postoperatively as a cutoff for early HRQOL assessment. The second subgroup analysis was based on the association between post-operative complications and HRQOL. Since no study had addressed HRQOL based on complications and no data were available to assess early versus late HRQOL, the subgroup analyses were not performed. 

## 4. Results

### 4.1. The literature Search

**Figure 1:**
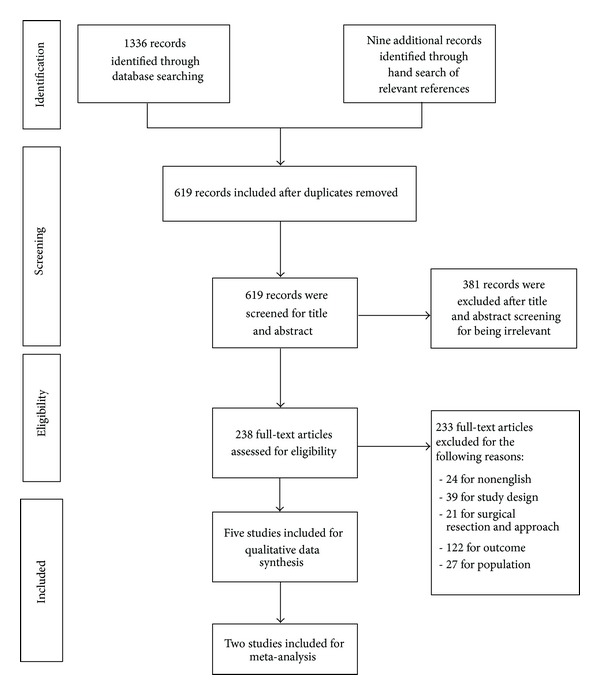
Flow chart summarizing the results of the screening process and study selection as per the PRISMA guideline.

Our database search yielded 1,336 records and hand search of relevant articles, and previous systematic reviews added 9 more records. After the removal of duplicates, 619 records were identified for the first step of the screening process. Through our initial titles and abstracts screen, 381 records were excluded. For the remaining 238 articles included in the full-text review, 233 articles were subsequently excluded (Figure 1).

Of the five articles that met the inclusion criteria, no randomized control trials were identified. One study was a cohort design, two were cross-sectional, and the remaining two were case series [10–14].

The five included studies in this review scored from 15 to 20 (out of 31) on the risk of bias assessment tool; most of the studies scored high on the power criterion based on the high number of subjects included in these studies. On reporting and selection of bias, all the studies scored an average or above average. Four of the studies failed to reach an average score on confounding, with the exception being Li et al., which scored 3 out of 6.  Table 1 summarizes the results of the risk of bias in the five studies included in this review.

### 4.2. Qualitative Review of Included Studies

Balduyck et al. conducted a cohort study in Belgium which included 100 patients in total. This study divided the patients into three groups based on the extent of resection, rather than the approach of surgery (group 1: lobectomy, group 2: pneumonectomy, and group 3: wedge resection). The study included patients with VATS and thoracotomy, although only one patient in the lobectomy group had a VATS resection, and no patients from the pneumonectomy group underwent VATS resections. The study used the European Organization for Research and Treatment of Cancer (EORTC) HROQL questionnaires QLQ30 and QLQ13. The researchers declared no conflict of interests [10]. Balduyck et al. concluded that the VATS group had a better HRQOL at three months after surgery mainly in physical functioning and thoracic pain as compared to thoracotomy. Also, the study demonstrated a better HRQOL in terms of bodily pain, global health, and physical functioning up to 12 months after surgery compared to thoracotomy.

Baysungur et al. conducted a cross sectional study in Turkey. The study had 18 patients in the VATS group and 20 patients in the thoracotomy group. HRQOL was assessed at 6 months after surgery using the EORTC QLQ 30 and QLQ 13 as well as the Health Survey SF-36 [11]. The results showed that the VATS group had improved HRQOL scores mainly in physical functioning and role limitation-emotional. The VATS group also scored better in the following symptoms: cough, neuropathy, chest pain and shoulder pain, and cognitive function.

Li et al. conducted a cross sectional study in Hong Kong. This study looked at HRQOL using the EORTC QLQ30 and QLQ 13. The median time after surgery when the questionnaire was administrated was 20.8 months for the VATS group and 37.7 months for the thoracotomy group. This study included 27 patients in the VATS group and 24 patients in the thoracotomy group. The researchers in this study did not report conflict of interests [14]. In this study, patients who had VATS resection tended to have better HRQOL, and the most commonly reported symptoms were cough, fatigue, thoracotomy pain, and dyspnea.

Brunelli et al. and Ilonen et al. reported on two case series that included only patients with thoracotomy. One hundred and fifty-six and 48 patients were included in each series, respectively. We decided to include these two studies in our qualitative analysis to describe the post-operative changes in the HRQOL of patients who had thoracotomy for lung resection for NSCLC. Ilonen et al. declared no conflict of interests, while Brunelli et al. did not report on conflict of interests [12, 13]. Tables 2 and 3 summarize the characteristics of the included studies.

In the Brunelli case series, a comparison of baseline HRQOL to post-operative open thoracotomy HRQOL was conducted 1 and 3 months after thoracotomy. The results of this study showed that the Physical Composite Score (PCS) of the SF-36 was lower than baseline at one month after surgery but returned to baseline score at three months after surgery. The PCS is a summary of four domains that include physical functioning, role limitation-physical, bodily pain, and general health. The other component of the SF-36 is the Mental Composite Score (MCS), which did not show any difference at one and three months after surgery when compared to baseline. This study also showed that patients with lung cancer have lower HRQOL measured by the SF-36 at baseline when compared to the general population.

In the case series of Ilonen et al., assessment of HRQOL at 3, 12, and 24 months after thoracotomy was compared to baseline HRQOL using a generic measure, the 15D questionnaire. The results of this study showed that patients with lung cancer have lower HRQOL scores when compared to the general population at baseline, specifically in the breathing, mental health, discomfort, and distress domains. This study also concluded that women report more depression symptoms at three months after surgery than men. Additionally, they found that men have lowered sexual function up to 12 months after surgery. A clinically significant decline in total 15D score was noted at 3, 12, and 24 months postoperatively compared to baseline. This occurred in the dimensions of mobility, breathing, usual activities, and sexual activity.

### 4.3. Meta-Analysis of Included Studies

The meta-analysis only included the two cross-sectional studies, because we could not extract data from the cohort study (as the groups were divided based on extent of resection, rather than surgical approach), and the two case series included patients with thoracotomy only. The reviewers attempted to contact the corresponding author of the cohort study to obtain the data required but were unsuccessful.

Pooled estimates from these two included studies had *I* ^2^ values ranging between 0 and 95% depending on the scale or symptom of HRQOL being assessed. When assessing scales of global health, role limitation, cognitive scale, physical scale, and emotional scale, the *I* ^2^ value was 0% to 40% indicating no to minimal heterogeneity between the studies. On the other hand, symptoms like chest pain, shoulder pain, and coughing had an *I* ^2^ value of more 90% indicating high heterogeneity between the two studies and were therefore precluded, *a priori*, from our reporting of pooled estimates.

**Figure 2:**
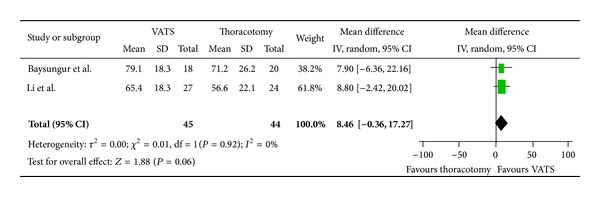
Forest plot for Global Health comparing VATS to thoracotomy.

**Figure 3:**
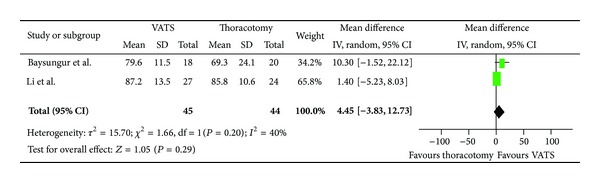
Forest plot for Physical scale comparing VATS to thoracotomy.

**Figure 4:**
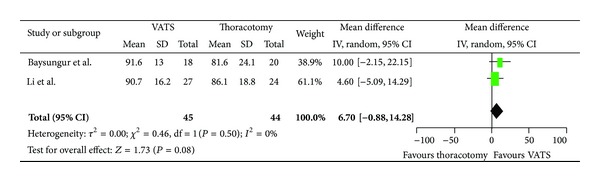
Forest plot for role limitation comparing VATS to thoracotomy.

**Figure 5:**
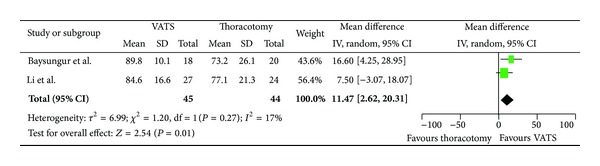
Forest plot for cognitive function scale comparing VATS to thoracotomy.

 Assessing global health, patients undergoing VATS resection had a mean improvement of 8.46 (95% CI, −0.36, 17.27) compared to patients who had undergone a thoracotomy resection (Figure 2). In regard to physical scale, patients with VATS resection had a mean improvement of 4.45 (95% CI, −3.83, 12.73) as compared to patients who had undergone thoracotomy (Figure 3). For role limitation scale, patients with VATS resection had a mean improvement of 6.7 (95% CI, −0.88, 14.28) as compared to patients who had undergone thoracotomy (Figure 4). Assessing cognitive scale, patients with VATS resection had a mean improvement of 11.47 (95% CI, 2.62, 18.07) as compared to patients undergoing thoracotomy (Figure 5).

### 4.4. Comments

In general, patients with NSCLC when compared to the general population have lower HRQOL indices preoperatively [12, 13]. Post-operatively, in patients undergoing thoracotomy there is an initial decline in their HRQOL (mainly in the physical component) that returns to baseline around three months after surgery [12]. 

Comparing VATS to thoracotomy, the VATS group have a better HRQOL scores that are seen up to 2 years after surgery (mostly related to physical health) [10, 11, 14]. Although the meta-analysis supported the qualitative analysis in favoring the VATS group in all the scales and symptoms, it also showed that most of these differences were not statistically significant.

To advance this field, and better inform clinical advice provided to patients undergoing surgical resection of NSCLC, we encourage the consistent use of a disease specific HRQOL instruments, such as the EORTC QLQ30/QLQ13. Based on the currently available evidence, the impact of such treatment approached on patient-reported outcomes is unclear.

One of the main limitations of this systematic review is the high risk of bias in the included observational studies. English only reports and publication bias are other limitations in this review, we did not assess for publication bias in this review due to the small number of studies included. Direct comparisons between studies were often limited because of (1) the variety of general and disease-specific HRQOL measures used, (2) the variability in NSCLC disease stage within populations, (3) the timing of the measurements of the HRQL instruments post-operatively, (4) the use of cointerventions such as chemotherapy and radiotherapy between studies, and (5) variable response rates and post-operative mortality resulting in potential selection bias.

Future research is required in this area to assess HRQOL between the VATS and thoracotomy approaches mainly in the early post-operative period. A high quality RCT or well-conducted observational studies are encouraged specially in North America.

## Figures and Tables

**Table 1:** Results of the risk of bias assessment using the Downs and Black assessment tool.

Study ID	Reporting (10)*	External validity (3)*	Bias (7)*	Confounding (6)*	Power (5)*	Total (31)*
Balduyck et al. [10]	5	1	5	2	5	18
Baysungur et al. [11]	6	0	5	2	4	17
Brunelli et al. [12]	5	1	5	0	4	15
Ilonen et al. [13]	5	1	4	2	4	16
Li et al. [14]	8	1	4	3	4	20

*Maximum number can be scored in that criterion.

**Table 2:** Summary of study characteristics.

Study ID/country	Time	Design	Intervention/comparison	HRQOL assessment (time of assessment in months)
Balduyck et al. [10]	2002–2004	Cohort	VATS/thoracotomy	QLQ30/13 (1, 3, 6, 12)

Baysungur et al. [11]	2007–2009	Cross sectional	VATS/thoracotomy	SF 36 QLQ30/13 (6)

Brunelli et al. Italy [12]	2004–2006	Case series	Thoracotomy only	SF 36 (1, 3)

Ilonen et al. Finland [13]	2002–2005	Case series	Thoracotomy only	15D (3, 12, 24)

Li et al. China [14]	1994–2000	Cross sectional	VATS/thoracotomy	QLQ30/13 (20.8 : 37.7)

**Table 3:** Summary of study characteristics (cont.).

Study ID	Population number (V : T)*	Mean age (V : T)* in years	Male sex % (V : T)*	Complications (V : T)*	COI^$^
Balduyck et al. [10]	100	CED^$$^	CED^$$^	NR**	No
Baysungur et al. [11]	(18 : 20)	(63 : 58)	(70% : 80%)	(1 : 1)	No
Brunelli et al. Italy [12]	156	65	79%	NR**	NR**
Ilonen et al. Finland [13]	48	63	62%	13	No
Li et al. China [14]	(27 : 24)	(63 : 66)	(74% : 75%)	NR**	NR**

V: VATS, T: Thoracotomy, ^$^COI: conflict of interests, ^$$^CED: could not extract data, **NR: not reported.

## Appendix

### Medline Search Strategy

1. lung neoplasms/or bronchial neoplasms/or carcinoma, bronchogenic/or carcinoma, non-small-cell lung/
2. (lung adj2 (cancer* or carcinoma* or tumo?r* or malignan*)).mp.
3. 1 or 2
4. thoracoscopy/or thoracic surgery, video-assisted/
5. (thora* or chest).mp. and (surg*.mp. or su.fs. ) and video*.mp.
6. thoracosco*.ti.
7. 4 or 5 or 6
8. 3 and 7
9. exp Questionnaires/or exp “Quality of Life”/
10. exp Health Status/
11. “Activities of Daily Living”/
12. health surveys/or exp population surveillance/
13. quality-adjusted life years/
14. treatment outcome/
15. Psychometrics/
16. px.fs.
17. “Outcome Assessment (Health Care)”/
18. (patient reported outcome* or quality of life or quality adjusted life year* or health state or health status or life quality or self report*).ti,ab.
19. (qol or hqol or hrqol or qaly).ti,ab.
20. (short form 12 or short form 36 or euroqol or quality of life questionnaire* or Quality of Wellbeing Index or Medical Outcomes Survey).ti,ab.
21. health utilit* index.ti,ab.
22. Health* year* equivalen*.ti,ab.
23. (endpoint* or end point*).ti,ab.
24. functional outcome*.ti,ab.
25. (health outcome* or outcome measure*).ti,ab.
26. (wellbeing or well being).ti,ab.
27. utilit*.ti.
28. or/9–27
29. 8 and 28.

## Abbreviations

CI: Confidence interval
EORTC: European organization for research and treatment of cancer
HRQOL: Health related quality of life
NSCLC: Non-small cell lung cancer
MCS: Mental composite score
MD: Mean difference
PCS: Physical composite score
RCT: Randomized control trial
SF-36: Short form-36
VATS: Video assisted thoracoscopic surgery.
